# Silver Diamine Fluoride in Pediatric Dentistry: Effectiveness in Preventing and Arresting Dental Caries—A Systematic Review

**DOI:** 10.3390/children11040499

**Published:** 2024-04-22

**Authors:** Alexandrina Muntean, Soundouss Myriam Mzoughi, Mariana Pacurar, Sebastian Candrea, Alessio Danilo Inchingolo, Angelo Michele Inchingolo, Laura Ferrante, Gianna Dipalma, Francesco Inchingolo, Andrea Palermo, Ioana Roxana Bordea

**Affiliations:** 1 Department of Paediatric Dentistry, Iuliu Hatieganu University of Medicine and Pharmacy, 31 A. Iancu Street, 400083 Cluj-Napoca, Romania; alexandrina.muntean@umfcluj.ro (A.M.); sounmyr@yahoo.fr (S.M.M.); sebastian.candrea@umfcluj.ro (S.C.); 2 Department of Orthodontics, Faculty of Dental Medicine, University of Medicine, Pharmacy Science and Technology “G. E. Palade” Targu Mures Romania, Gheorghe Marinescu Street, nr. 38, 540139 Târgu Mureș, Romania; mariana.pacurar@umfst.ro; 3 Department of Interdisciplinary Medicine, University of Bari “Aldo Moro”, 70124 Bari, Italy; a.inchingolo1@studenti.uniba.it (A.D.I.); a.inchingolo3@studenti.uniba.it (A.M.I.); laura.ferrante@uniba.it (L.F.); gianna.dipalma@uniba.it (G.D.); 4 College of Medicine and Dentistry, Birmingham B4 6BN, UK; andrea.palermo2004@libero.it; 5 Department of Oral Health, Iuliu Hatieganu University of Medicine and Pharmacy, 15 V. Babes Street, 400012 Cluj-Napoca, Romania; bordea.ioana@umfcluj.ro

**Keywords:** silver diamine fluoride, dental caries, prevention, caries arrest, cariostatic agents, pediatric dentistry

## Abstract

Background: Tooth decay is considered a global scourge by the World Health Organization (WHO) starting at an early age. In recent years, silver diamine fluoride (SDF) has regained interest, particularly in pediatric dentistry, used to prevent the development of carious lesions or arrest their progression. Objective: The aim of this study was to assess, through a systematic review of the literature, the effectiveness of SDF, used in pedodontics, in temporary teeth, in preventing or arresting dental caries. Material and Methods: An electronic search was conducted on PubMed, Web of Science and Scopus. The effect of SDF on both temporary and permanent teeth has been considered. Results: The inclusion criteria identified 16 randomized controlled trials involving patients aged 18 months to 13 years and followed over a period of 12–30 months. Conclusions: SDF is a practical, accessible and effective non-invasive way to prevent and arrest caries in temporary and permanent teeth. Its application requires regular monitoring. The resulting black spot is diminished by immediate application of potassium iodide but this may affect its effectiveness.

## 1. Introduction

Dental caries, the prevalent chronic condition observed in children worldwide, arises from a complex interplay of factors. Dental susceptibility, compounded by temporal progression, heightens individuals’ vulnerability to this pathology. Additionally, suboptimal oral hygiene practices, compounded by poor dietary habits, create an environment conducive to the proliferation of biofilm bacteria, thereby exacerbating the risk of caries onset. These multifaceted determinants collectively underscore the intricate nature of addressing this pervasive oral health issue [1,2].

In the absence of treatment, the carious process progresses to reach the pulp and leads to a periapical periodontitis abscess—or an endodontic—periodontal involvement such as parulis in the furcation area. Simple and complicated decay of primary and immature permanent teeth is a common pathology in children, due to the morphological and structural peculiarities of these teeth. It should be mentioned that, in the particular case of Romania, where there are no national dental caries prevention programs, this pathology has an increased incidence [3,4,5,6,7].

Fluoride agents applied directly to carious lesions can slow down the carious process and convert an active lesion into an inactive one [8,9,10]. Recently, there has been an upsurge in the use of silver fluoride products such as silver diamine fluoride (SDF) which was first promoted in Japan in the late 1960s by Dr. Yamaga and Dr. Nishino to arrest dental caries [11,12,13,14,15,16,17,18,19,20,21,22,23,24,25,26].

SDF is most frequently used at a concentration of 38%. AgFH6N2 at 24% to 27% silver is used for its antimicrobial effect on *Streptococcus mutans* and *Lactobacillus acidophilus* [27], so silver microwires have been described in teeth previously treated with SDF, which would replace the defects resulting from demineralization by the carious lesion [28]. Using 5 to 6% fluoride replaces the hydroxyl group with fluoride in hydroxyapatite, and demineralization is inhibited [29]. Alkaline ammonia solution (7.5 to 11%) is used to stabilize all ingredients [5,30,31,32,33,34,35].

It has also been indicated that SDF has a greater inhibitory potential on matrix metalloproteinases (enzymes responsible for the degradation of pericellular substrates [36]) than sodium fluoride (NaF) and silver nitrate (AgNO3) [11,37,38].

Due to its accessibility and its ease and speed of use, SDF represents an advantage, compared to conventional restorative treatments, in case of behavioral and/or economic difficulties [39,40]. SDF has also seen renewed interest since early 2020, with the COVID-19 health crisis, as an alternative to aerosol-generating procedures [41,42].

## 2. Materials and Methods

An electronic search, conducted until 25 April 2023, encompassed databases such as PubMed^®^, Web of Science, and Scopus, employing Mesh terms to pinpoint randomized controlled trials examining the efficacy of silver fluoride diamine in halting or preventing dental caries in children and young adolescents. Boolean equations were formulated to refine the search parameters. The first equation targeted publications related to silver diamine fluoride, yielding 485 results. The second equation focused on pediatric dentistry, children, and adolescents, ensuring the comprehensive coverage of relevant literature in the field. 

### 2.1. Inclusion Criteria

Randomized controlled clinical trials were included if published from 1 January 2012, written in English, French or Romanian and involving human subjects, with a follow-up of at least 6 months. Also, for an article to be included in this study, a full-text version of the paper had to be available.

Data extraction involved utilizing an analysis grid to systematically retrieve relevant information from the studies. Key data points included details on the study population and any control groups, such as the country of origin, sample size, and age demographics. Additionally, information regarding the dental condition of participants was extracted, covering aspects such as the type of dentition, prevalence of dental caries (including dmft/DMFT scores), dental sensitivity, and any other noted dental pathologies.

Moreover, the extraction process involved capturing specifics regarding the application of silver diamine fluoride (SDF) or other substances, including indications for use, concentration, method of application (whether on temporary or permanent teeth), duration of exposure to SDF, frequency of applications, and intervals between treatments. Furthermore, details regarding the materials utilized for cavity restoration post-treatment were recorded.

Finally, the duration of the follow-up period post-treatment was noted, along with documentation of any instances of arrested or unarrested caries observed during this period.

This study was registered in Prospero with the ID CRD42023451353.

### 2.2. PICO

Population: Children and young teenagers with dental caries or at risk of developing dental caries.

Intervention: Use of silver diamine fluoride (SDF) for preventing or arresting dental caries.

Comparison: Comparisons vary depending on the specific study but may include placebos, other fluoride agents, or conventional restorative treatments.

Outcome: Effectiveness of SDF in preventing or arresting dental caries, as measured by various parameters such as decay progression, caries arrest rates, hypersensitivity scores, and other clinical outcomes.

## 3. Results

### 3.1. Study Selectionm

Among the 188 previously identified publications, 18 articles were excluded before reading:

- 11 published before 2012,
- 1 written in German, and
- 6 PDF not available.

And 149 after reading:

- 61 not responding to the object of this review.
- 66 presenting a study design other than a randomized controlled trial, including 21 systematic reviews, 12 narrative reviews, 8 cohort studies, 4 umbrella reviews, 5 commentary publications, 2 non-randomized comparative studies, 2 cross-sectional studies, 2 overviews, 2 guidelines, 1 case report, 1 pilot study, 1 retrospective study, 1 Delphi study, 1 letter to the editor, 1 selection of abstracts, 1 critical summary and 1 promotional publication.
- 9 study protocols not carried out on patients, 5 studies whose follow-up period was extended in randomized controlled trials selected for this study, 4 studies whose follow-up period is less than 6 months, 2 publications relating to animals and 2 in vivo studies.
- All this information is part of the Prisma flow chart.

Selection process: three authors (A.M., S.M.M., and M.P.) screened titles independently and compared their findings. Study selection was assessed independently by two authors (A.M. and M.M.), who performed the assessment of titles and/or abstracts of retrieved studies. In cases of disagreement, studies were included after consensus was reached through discussion between the two mentioned authors and a third author (M.P.).

#### Selected Studies

The inclusion criteria adopted allowed the selection of 23 randomized controlled trials, involving patients aged 18 months to 13 years and followed over a period of 12–30 months.

Three-quarters of the included studies come from Asia, Hong Kong being the region to have conducted the most clinical trials (5 trials [8,43,44,45,46]) followed by Thailand (3 trials [47,48,49]), China [50], Saudi Arabia [51], Cambodia [11], India [52,53] and the Philippines [54] (1 trial each).

In Brazil, two studies explored the effectiveness of 30% SDF in arresting caries affecting molars [55,56]. In Turkey, the study performed by Ballikaya et al., investigated the potential of 38% SDF in the treatment of incipient carious lesions of permanent molars affected by hypomineralization within the syndrome of molars and incisors hypomineralization (MIH) [57].

Among the selected studies, one was conducted in Michigan in the United States, and another in New York, where SDF has been available there since April 2015 after being authorized for marketing by the Food and Drug Administration. According to dentition, 15 of the included studies focused on primary teeth, involving the majority of patients versus 3 studies on permanent teeth [58,59,60,61].

Regarding the treatment of the patients SDF was compared with either placebo or with classic therapies being part minimally invasive dentistry (MID). The protocol applied was with minor variations being based on cleaning and drying the tooth surface before SDF application; application for 10–60 s; followed by wiping with a gauge and finishing with the indication of food restriction (30–60 min) [54,62].

**Figure 1:**
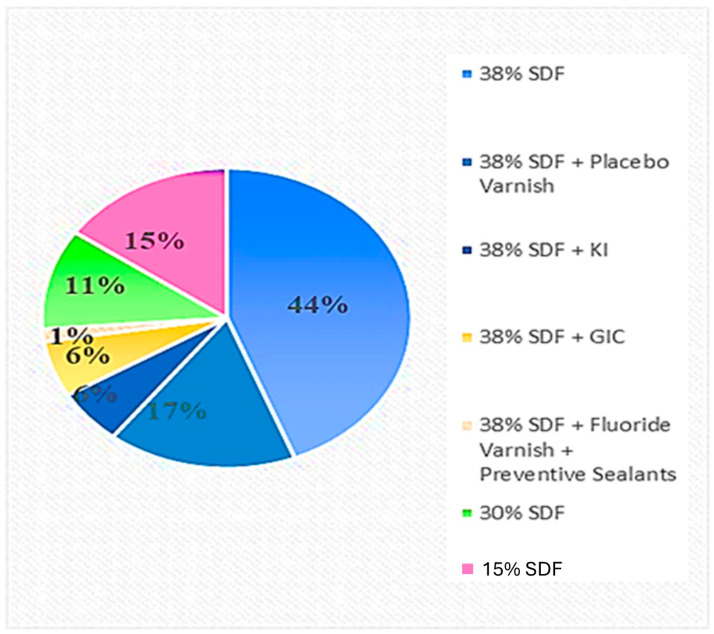
SDF treatment protocols.

In order to evaluate SDF effectiveness authors proposed different associations: KI, glass-ionomer cements (GIC), fluoride varnish or dental sealants (Figure 1). 

**Figure 2:**
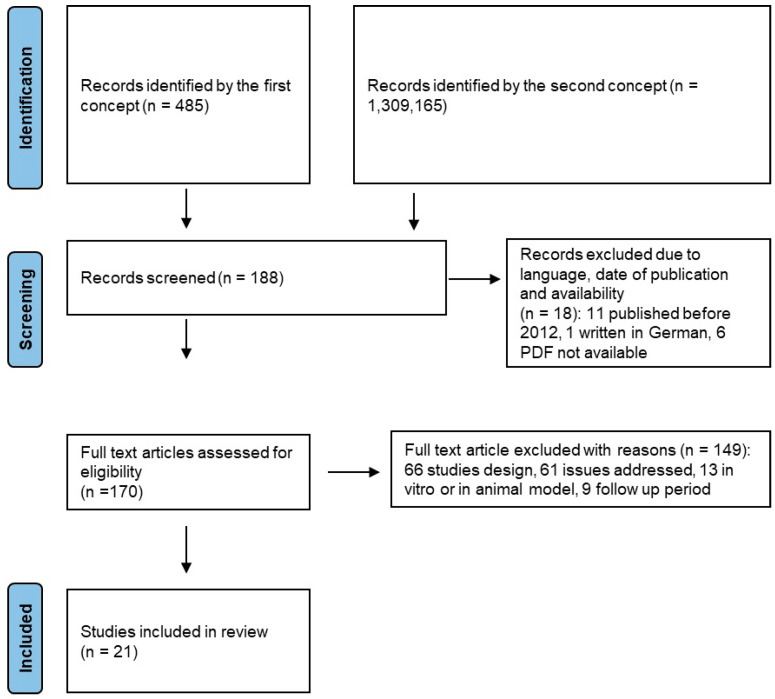
Prisma flow chart.

Figure 2 shows the PRISMA flow chart illustrating the path of information through the different stages of research.

The quality of the included papers was assessed by two reviewers, R.F. and E.I., using the ROBINS is a tool developed to assess risk of bias in the results of non-randomized studies that compare health effects of two or more interventions. Seven points were evaluated and each was assigned a degree of bias. A third reviewer (F.I.) was consulted in the event of a disagreement until an agreement was reached. The question in the domains evaluated in the ROBINS is the following:

- Bias due to confounding,
- Bias arising from measurement of exposure,
- Bias in the selection of participants into the study,
- Bias due to post-exposure intervention,
- Bias due to missing data,
- Bias arising from measurement of the outcome, and
- Bias in the selection of the reported results.

### 3.2. Quality Assessment and Risk of Bias of Included Articles

**Figure 3:**
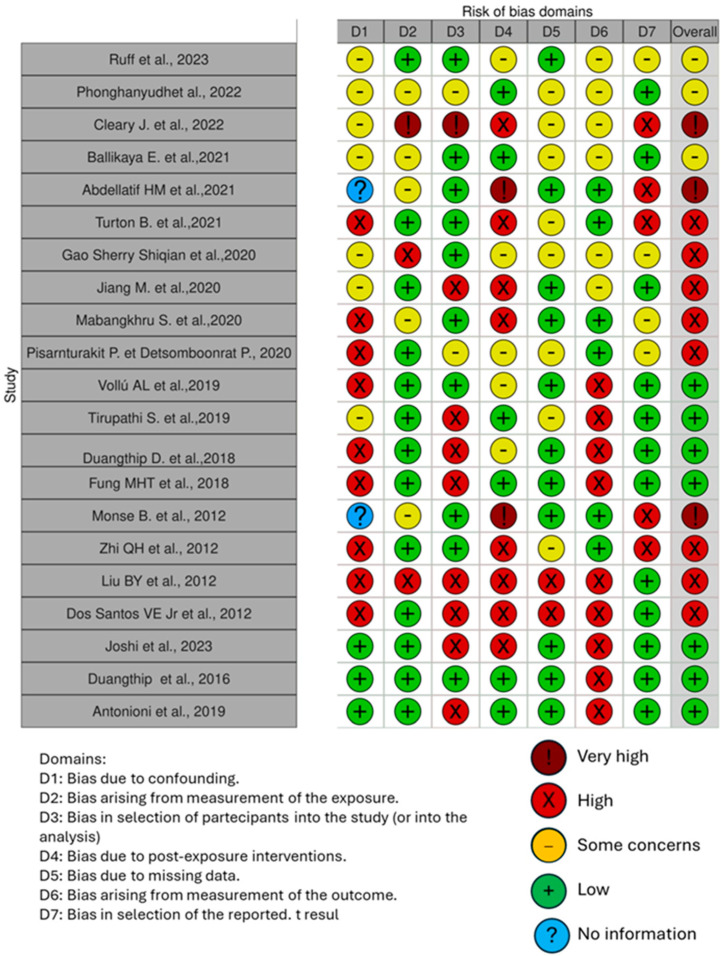
Bias assessment [8,11,43,44,45,46,47,48,49,50,51,52,53,54,55,56,57,58,60,61,64].

The risk of bias in the included studies is reported in Figure 3. Regarding the bias due to confounding most studies have a high risk. The bias arising from measurement is a parameter with low risk of bias. Many studies have a low risk of bias due to bias in selection of participants. Bias due to post exposure cannot be calculated due to high heterogeneity. The bias due to missing data is low in many studies. Bias arising from measurement of the outcome is low. Bias in the selection of the reported results is high in most studies. The final results show that eight studies have a high risk of bias, two have a very high risk of bias and four have a low risk of bias [63].

## 4. Discussion

### 4.1. Permanent Teeth

During repeated applications of Riva Star© 38% SDF at 1, 6 and 12 months involving 106 permanent molars with MIH hypomineralization and ICDAS lesions 1–2, Ballikaya E. et al., 2021, did not note a significant difference in hypersensitivity scores between the group who received, following the application of SDF, potassium iodide (KI) and the one who participated to an atraumatic restorative treatment modified with silver and accompanied by a hybrid glass ionomer cement (GIC) filling. Nevertheless, the results were more promising for the occlusal surfaces than for the palatine surfaces where the cumulative efficiency rates were 88.7% and 58.8% [57,65].

Monse B et al. 2012, indicated 38% SDF to prevent carious lesions affecting the dentin (D3) of the occlusal surfaces of the first permanent molars (*n* = 706), in 230 children aged 6 to 8 years. In order to assesses SDF effectiveness children were divided into two groups; for the first group (*n* = 139) at the end of 18-month follow-up, a single application of 38% SDF was 87.5% effective in decay progression, against a rate of 91% in the second group (*n* = 91) where children brushing with a fluoridated toothpaste [66]. The results demonstrate the SDF effectiveness and allow to consider it as a method for decay prevention/arresting in non-cooperative or special care need children [54].

Liu BY et al. 2012, carry out an annual application of 38% SDF in 121 participants in with the purpose to prevent pits and fissures caries; 378 permanent molars were included and an 87.8% efficiency rate result after 2 years monitoring [46].

### 4.2. Temporary Teeth

The study by Cleary J. et al., 2022, started with 98 children with an average age of 4.8 (1.8) years and ended with 68 after 12 months follow-up. One random lesion per child received 38% SDF (twice, at a 6-mo interval) or restorative treatment. Authors considered preventive effect and clinical failure rates as follows: minor (e.g., reversible pulpitis, active/soft lesion or progression, restoration loss or need for replacement/repair, secondary caries) and major (e.g., irreversible pulpitis, abscess, extraction) [24,58]. The effectiveness of 38% SDF in arresting caries at 74% was associated with minor failures (65%) as well as major failures (13%), against 23% of minor failures and 3% of major failures in the group that received conventional restorative treatment. However, it is admitted that the application of SDF was easier and faster [58].

The trial conducted by Abdellatif HM et al., 2021, in 53 patients (SDF group = 27 children) with a mean age of 5.7 (1.2) years and a mean dmft score of 4.13 (1.92), is the one that reported the greatest efficacy of SDF at 12 months where 99% of identified carious lesions were arrested [51].

Turton B. et al., 2021, attained better results with SDF (77.3% and 65.4%) than silver fluoride AgF (75.3% and 51.2%) for arresting lesions (ICDAS 3–4 and 5–6) affecting deciduous teeth in a child population with a mean age of 7.6 (1.9) years.

The recourse to potassium iodide KI, following the application of SDF or AgF, offered a better aesthetic result but a poorer caries control [11].

Potassium iodide (KI) is often used in dentistry in conjunction with agents such as silver fluoride (AgF) or silver diamine fluoride (SDF). This combination is employed to treat carious lesions, especially on the anterior surfaces of teeth, where aesthetic appearance is particularly important [10,11].

The role of KI is twofold. Firstly, it helps reduce the discoloration caused by the presence of silver compounds after treatment with AgF or SDF. This is significant because discoloration can be an undesirable side effect of the treatment and can negatively impact the appearance of the teeth, especially in pediatric patients [10,11].

However, there is a trade-off. Removing silver compounds to reduce discoloration may compromise the effectiveness in caries control. Indeed, silver ions have antibacterial properties that contribute to caries prevention. So, while KI improves the aesthetics of treated teeth, it may reduce effectiveness in preventing the formation of new carious lesions. This balance between aesthetics and caries prevention is important to consider in clinical practice [10,11].

While KI has been proposed as a means to reduce the discoloration associated with SDF treatment, its effectiveness in this regard has been met with conflicting evidence. Some studies suggest that the application of KI after SDF treatment may help minimize staining of the treated teeth, thus addressing a common concern among patients and caregivers. However, other research indicates that the addition of KI may not significantly reduce discoloration or may even exacerbate it in some cases [10,11].

Furthermore, the use of KI with SDF raises questions about its impact on the caries-controlling properties of SDF. Some studies have suggested that the addition of KI may compromise the antimicrobial efficacy of SDF, potentially diminishing its ability to arrest and prevent dental caries. This underscores the need for careful consideration of the potential trade-offs associated with using KI in conjunction with SDF [10,11].

In light of this conflicting evidence, it is essential for clinicians to weigh the potential benefits of reducing staining against the possible impact on the caries-controlling properties of SDF when considering the use of KI. Additionally, further research is needed to elucidate the optimal protocol for incorporating KI into SDF treatment regimens and to better understand its effects on both esthetics and caries management. The trial conducted by Gao Sherry Shiqian et al., 2020 [43], is the only clinical trial to have explored, over a period of 30 months, the potential of a semi-annual application of 38% SDF (combined with a placebo varnish) to arrest early childhood caries by comparing it with that of the association of silver nitrate 25% AgNO3 and sodium fluoride 5% NaF.

Equivalent efficacy rates for 38% SDF (68.9% and 60% at, respectively, 30 and 12 months) and the association of AgNO3 25% and NaF 5% (70.6% and 62.4%, respectively) were highlighted at, respectively, 30 and 12 months) [43].

The youngest population relates to children aged 1 to 3 years (mean age: 36.8 (6.4) months) and was studied by Mabangkhru S. et al., 2020. These high caries risk children, presenting an average dmft score of 5.3 (3.6), received 38% SDF (Group 1) for 10 s by means of a disposable micro-applicator arresting 35.7% of the carious lesions. Although this is a relatively low rate, it is still higher than that of Group 2 (20.9%) to which a 5% NaF varnish was applied [47,67]. 

Pisarnturakit P. and Detsomboonrat P., 2020 divided 89 children aged 3 to 5 years into 3 groups; 2 groups, one at high risk (HRB), the second at low risk (LRB) benefited from basic prophylactic care (Table 1), 1 other high-risk group (HRI) received, in addition to prophylactic means, 38% SDF followed by fluoride varnish application.

The percentages of patients who developed new caries were mentioned as follows:

- HRI group: 65.7%, 5% and 40% at 24, 12 and 6 months, respectively.
- HRB group: 75%, 42.9% and 45.9% at 24, 12 and 6 months, respectively.
- LRB group: 21.1%, 10.5% and 5.3% at 24, 12 and 6 months, respectively.

Nonetheless, for the HRI group, the active lesion of 138 out of 159 surfaces (86.8%) became arrested caries after SDF treatment [48,68].

Fung MHT et al., 2018 indicated a better efficacy of 38% SDF when applied twice a year, estimating it at 75.7% and 62.6% (at 30 and 12 months, respectively) while the efficacy of the 38% SDF applied only once a year has been estimated at 66.9% and 51.9% (at 30 and 12 months, respectively) in young children aged 3 to 4 years at the beginning of the study [5,45].

Similarly, Zhi QH et al., 2012 showed that, in a population of the same age group with a mean dmft score of 5.1 ± 4.0 and followed for 2 years, 38% SDF was more effective to arrest dentin caries when applied semi-annually (90.7% at 24 months) rather than once a year (79.2% at 24 months). Moreover, after the first applications of 38% SDF, four evaluations were made (at 6, 12, 18 and 24 months) highlighting the continuous increase in its efficacy rate. Indeed, its effectiveness more than doubled between the 6th and 24th month [50].

Tirupathi S. et al., 2019 considered that an annual application 38% SDF (Saforide) has the same clinical efficacy (71.05%) as Nano-Silver-incorporated Sodium Fluoride 5% NSSF (77%) in the prevention of the progression of dentinal caries affecting the posterior deciduous teeth, in children aged 6 to 10 years with an average dmft score of 4.35 ± 2.15 (76 lesions) for the SDF group and 4.67 ± 1.76 (71 lesions) for the NSSF group [52].

Along with Mabangkhru S. et al., 2020, Jiang M. et al., 2020 applied 38% SDF for 10 s to 88 children aged 3 to 4 years with a mean dmft score of 4.6 ± 3.6. They also reported a fairly low rate of SDF’s effectiveness despite the pre-treated cavity having been restored with a glass ionomer cement and found, contrary to Zhi QH et al., 2012, a decreasing SDF’s effectiveness each semester [50].

### 4.3. 30% SDF

Vollú AL et al., 2019 and Dos Santos VE Jr et al., 2012 applied 30% SDF (Cariestop Biodynamic) to temporary molars. Although the results obtained by Dos Santos VE Jr et al., 2012 related to the efficacy of 30% SDF on ICDAS 5 lesions (66.9% at 12 months and 84.7% at 6 months), among 48 children aged 5 to 6 years, are more satisfactory than those mentioned for the group that received an Interim Therapeutic Restoration (IRT) with the help of a glass ionomer cement GIC (38.6% at 12 months and 53.1% at 6 months), they remain lower than the efficacy rate indicated by Vollú AL et al., 2019 (84.6% at 12 months) and achieved thanks to a semi-annual application of 30% SDF to which 65 molars were exposed for 3 min among 34 younger children, aged 2 to 5 years [55,56].

Duangthip D. et al., 2018 noted, at the end of the 30-month follow-up period, similar results regarding the annual use of 30% SDF (45%) or its recourse in 3 applications at weekly intervals (44%) for the arrest of ICDAS 3–4 lesions in children aged 3 to 4 years who present an average dmft score of 3.7 ± 3.5 [6,47].

On the other hand, with regard to ICDAS 5–6 lesions, the annual recourse to 30% SDF turned out to be more effective (48%) than the three applications at weekly intervals (33%) [44,69].

### 4.4. 12% SDF

Fung MHT et al., 2018 estimated the rate of caries arrest by an annual application of a 12% SDF at 55.2% and 40.6% (at 30 and 12 months, respectively), while its biannual application arrested 58.6% and 48% of decays (at 30 and 12 months, respectively) [70]. These rates remain lower than those obtained among participants of the same study who received the 38% SDF [45].

Dental caries is a widespread problem worldwide and represents a significant financial burden [53]. The untreated carious lesions can have a great impact on the life of a young person by affecting the general health, throw systemic infections, as well as altering the social and school performance of the child [71,72,73]. 

SDF is considered an inexpensive treatment that addresses problems of access to dental care for disadvantaged groups. It is a topically applied liquid that can stop carious lesions without removing infected soft tissue [61]. It is emphasized that SDF is easier to apply than the ART (atraumatic restorative treatment) technique, making it accessible even to the general dentist with less technique or practice required.

The average caries arrest rate in the short term was higher than in previous studies, with a high success rate. Application of SDF with a half-yearly interval showed higher arrest rates than longer intervals.

SDF treatment is a clinically superior option compared with ART for stopping caries lesions. Some advantages include less time in the patient’s chair, lower costs, and less dependence on operator skills. Dental anxiety in children was assessed and no significant differences emerged between the SDF and ART groups. Parental satisfaction with cosmetic appearance showed no substantial differences, with little concern about color change in SDF-treated teeth [55].

SDF meets the WHO Millennium Goals as a simple, non-invasive, less technically sensitive, and cost-effective treatment for caries arrest [52]. SDF is particularly practical in children who are too young to tolerate restorative procedures very little [8,74] or no discomfort being noticed by the young patients and comes along with good acceptance [75]. SDF can be a put in the armamentarium available to dentists as a part of minimally invasive dentistry (MID)—concept being non-invasive, easy to apply and cost efficient in the same time being an accessible therapy in the pandemic times of COVID-19 [76,77,78]. MID strategies are orientated towards repairment rather than replacement of affected dental structures thus stopping carious advancement [79,80,81].

The collected data suggest that the 38% SDF solution is effective in preventing and arresting carious enamel and dentin lesions affecting permanent molars, including those with structural enamel defects such as MIH, in patients aged 1 to 13 years [49,57,82]. SDF had a preventive effect on 6-year-old molars with MIH; no new carious lesions appeared after SDF application) [36].

This idea is found in the literature with a question mark about the SDF efficiency as a prophylactic and therapeutic agent in the carious disease for the permanent dentition due to the lack of studies in this direction, although some studies suggest that SDF can be applied with or without dentinal removal, its penetrability not being affected by the carious dentin left in the cavity [83]. Seifo et.al concluded theirs review about SDF as a solution for caries in the above mentioned way [55,84,85].

As for deciduous teeth, SDF has been shown in numerous studies to prevent and arrest deep carious lesions in high caries risk children [86,87,88,89]. SDF at 38% can be used efficiently to arrest caries in deciduous teeth. It was shown in a recent study by Keyur H Joshi et al. in 2023 to be effective in changing the color and texture of carious lesions, with 92% of lesions considered arrested after 6 months [53,90].

Mabangkhru S. et al., 2020 and Duangthip D. et al., 2018 proved that 38% SDF and 30% SDF were more effective than 5% NaF varnish in arresting carious lesions in young children [44,47]. Zaffarano et al. concluded in the same idea in comparison with ART and fluoride in his review [91].

A significant difference between the use of 30% SDF and Interim Therapeutic Restoration with GIC was also noticed by Dos Santos VE Jr et al., 2012, attributing a higher rate of effectiveness to 30% SDF (66.9%) than to IRT with GIC (38.6%) [56]. Other reviews of literature conclude that there is no difference in caries arrest between SDF and GIC therapy [87,92].

The best results regarded biannual applications of 38% SDF [64], arresting up to 99% of initial carious lesions when assessed at 12 months [51]. Zhi QH et al., 2012 have also highlighted the interest of this six-monthly application in young children, obtaining an increasing efficiency rate every 6 months [50,82].

This semi-annual recourse to 38% SDF does not necessarily prevent the clinician from minor or even major failures, especially in high caries risk children, suggesting that the lesions treated by SDF must be correctly monitored [57,93].

The least satisfactory efficacy rates were found in the following situations: application of 38% SDF for 10 s [8,47].

In this regard, Marília-Franco Punhagui et al., 2021 appeal to an application time of one minute to promote enamel remineralization whether the SDF concentration is 30% or 38% [94]. Applications at weekly intervals of 30% SDF on ICDAS 5–6 lesions [44] and annual application of 12% SDF [45,95]. Moderate caries lesions showed no significant differences among the three treatment groups. The presence of plaque and the location of the lesion influenced treatment efficacy. Annual application of 30% SDF was more effective in stopping cavity dentinal caries lesions than weekly applications of SDF or fluoride varnish [96,97]. There were no significant differences in the treatment of moderate caries lesions between the different fluoride protocols. The study suggests that annual application of 30% SDF might be a promising approach to stop dentinal cavity caries lesions in preschool children with high caries risk [51,98].

The literature provides data about the effect of SDF on the most notorious cariogenic bacteria [99,100]. *Streptococcus mutans* (*S. mutans*) and lactobacilli experience an increase in metabolic activity in the presence of fermentable carbohydrates, thus being able to produce acids tilting the balance between dental mineral loss and gain towards the first [78,82,101,102]. SDF was able to significantly reduce the activity of the upper mentioned bacteria at one month survey according to Ammar et al. [99,103]. SDF manages to reduce the bacterial biofilm, not just the targeted pathogens [104,105,106].

As the appearance of blackish spots may constitute a discomfort for the patient and/or the parents, it is possible to proceed to an application of potassium iodide (KI) immediately after the use of the SDF in order to reduce this blackish appearance, yet this may interfere with caries control [107]. In this matter an in vitro study shows that the penetration of *S. mutans* is reduced in the silver fluoride and potassium iodine treated dentine [98,100].

For controlling the down side of SDF application several methods are suggested in the literature and they vary from silver nanoparticle and silver fluoride nanoparticles [103,108]; used to arrest carious lesions; to different remineralizing agents such as hydroxyapatite nanoparticles; or even using a complete different composition for carious arrest such as selenium nanoparticles which could present anti-staining properties [105,109]. In the study of Duangthip et al. in 2016, 304 children with active dentinal caries divided into three groups: Group 1 receives 30% silver diamine fluoride (SDF) annually, Group 2 receives three weekly applications of 30% SDF, and Group 3 receives three weekly applications of 5% sodium fluoride (NaF) varnish [108]. Follow-up at 18 months reveals that both SDF protocols are more effective than intensive NaF varnish applications in stopping caries [109]. Survival analysis indicates that SDF significantly reduces the time to caries arrest compared with NaF varnish, with no significant differences between the SDF protocols. The results support the use of SDF, emphasizing its effectiveness in arresting dentinal caries in high-risk preschool children [110].

In addition to clinical therapies such as the application of silver fluoride solutions (SDF), the prevention of dental caries and the maintenance of email health are crucial in the context of general dental health. In this context, the role of toothpastes is of paramount importance, as they represent a daily oral hygiene tool. Recent research has investigated the effects of different toothpaste formulations, including those containing hydroxyapatite, fluoride and their combinations, on human email in vitro.

Therefore, the study of Guntermann et al. [111] aimed to assess the remineralization potential and protection against demineralization of enamel by comparing a hydroxyapatite-containing toothpaste (Karex) with fluoride-containing (Elmex) and fluoride- and hydroxyapatite-free toothpaste (Ajona) as control. Enamel samples were exposed to acid to induce demineralization, then treated with respective toothpaste and evaluated using scanning electron microscopy. Elmex exhibited the lowest demineralization percentage (mean 5.01%), outperforming Ajona (8.89%) and Karex (9.85%). Elmex also showed the least demineralized enamel after new acid exposure (median 6.29%), followed by Ajona (11.92%) and Karex (13.46%). Results indicate Elmex’s superior remineralization and protection against renewed demineralization compared to Karex and Ajona. Karex’s lower efficacy may be attributed to micro-HAP particles’ limited penetration into enamel lesions. Other factors such as pH value and presence of zinc and xylitol may also influence toothpaste efficacy. Overall, the findings question the recommendation to use Karex for enamel protection, highlighting Elmex’s superiority in this aspect. Further studies are warranted to explore factors influencing toothpaste efficacy comprehensively [111].

## 5. Limitations

The limitations of the present study are among the critical aspects in the study design of the selected papers—there is the use of the dmft and DMFT indexes for caries assessment; the indexes are still a valid resource for epidemiological purposes but do not provide caries severity information as they are only dichotomous (presence/absence of caries) [112]. 

The deficiency of the standardization of the SDF protocol application emerged from all studies, potentially affecting their outcomes [91,113]. In addition, the setting procedures differ among the studies; if an effective isolation is not obtained, the effectiveness of the therapy can be impaired or limited. Another limitation is that only articles in English, French or Romanian were included in this review. Further studies are necessary to complete the results [54,114].

Heterogeneity: The studies varied in terms of patient demographics, study designs, intervention protocols, and outcome measures, leading to significant heterogeneity. This diversity could complicate the synthesis and interpretation of the results and limit the ability to draw definitive conclusions.

Duration of Follow-Up: Although the inclusion criteria specified a minimum follow-up period of six months, the duration of follow-up across the selected studies ranged from 12 to 30 months. This variability in follow-up duration could influence the assessment of long-term effectiveness and outcomes related to silver diamine fluoride (SDF) treatment.

Sample Size and Generalizability: Some studies had relatively small sample sizes or focused on specific patient populations, potentially limiting the generalizability of the findings to broader populations or clinical settings.

## 6. Conclusions

In conclusion, dental caries remains a prevalent and substantial concern globally, bearing significant implications for individuals’ general health, social dynamics, and educational attainment, particularly among young populations. Silver diamine fluoride (SDF) emerges as a promising intervention for caries prevention and arrest, particularly in pediatric dentistry settings, where conventional restorative approaches may be challenging. The efficacy of SDF in halting carious lesions, both in primary and permanent dentition, has been underscored across numerous studies. Notably, SDF exhibits considerable success rates in arresting caries progression, with biannual applications yielding superior outcomes compared to longer intervals. Moreover, SDF demonstrates advantages over conventional treatments such as the atraumatic restorative treatment (ART), boasting shorter chair times, reduced costs, and lower operator skill dependencies. Importantly, SDF aligns with the principles of minimally invasive dentistry (MID), offering a non-invasive, accessible, and cost-effective alternative, particularly vital amidst the challenges posed by the COVID-19 pandemic. However, challenges persist, including variations in application protocols and the emergence of black discoloration, prompting ongoing research into refining SDF formulations and application techniques. The standardization of SDF protocols and further investigation into its long-term efficacy and safety are imperative to maximize its potential as a cornerstone of modern caries management strategies. For optimal results, it can be applied twice a year at a concentration of 38% and subject to a regular monitoring. To overcome aesthetic discomfort, the resulting dark spot is reduced by applying potassium iodide although the SDF’s effectiveness may be reduced. Additionally, comprehensive assessments considering factors beyond the dmft/DMFT indexes and encompassing diverse linguistic literature will enrich our understanding of SDF’s utility in diverse clinical contexts. In essence, SDF stands as a valuable asset in the armamentarium of contemporary dentistry, offering a potent tool for curbing the pervasive burden of dental caries, especially in vulnerable populations. 

## Figures and Tables

**Table 1:** Articles included [8,11,43,44,45,46,47,48,49,50,51,52,53,54,55,56,57,58,60,61,64].

Study/ Region	Age Range	Treated Teeth	Follow Up/ Months	SDF Group(s)/ Sample Size	Other Group(s)/ Sample Size	Effectiveness of SDF in Arresting/ Preventing Decays	Effectiveness of Other Materials in Arresting/ Preventing Decays
Ruff et al., 2023 United States [60]	5–13 years	Primary and permanent teeth	24 months	38% SDF/413 patients	NA	Arrest rate for the experimental treatment was considerably higher than for the active control-(80%)	50%
Phonghanyudh et al., 2022 Thailand [49]	1–3 years	Primary teeth	18 months	147 patieints	143 patients	59.1%	58.1%
Cleary J. et al., 2022 United States [58]	2–10 years	1 randomly selected primary tooth per child, with at least 1/3 of the crown remaining, no signs of periapical infection and whose anticipated exfoliation > 12 months	12 months	38% SDF (2× per year)/ 40 patients	Conventional restorative treatment; Material selected, following AAPD guidelines 29 patients	At 12 months: 74% At 6 months: 57% Minor failures: 65% Major failures: 13%	Minor failures: 23% Major failures: 3%
Ballikaya E. et al., 2021 Turkey [57]	6–13 years	Permanent molars; -at least 2 first permanent molars that have erupted -MIH/hypomineralization -ICDAS lesions 1–2	12 months	Group 1: 38%SDF (2× per year)/45 patients Group 2: 38% SDF + ART (SMART)/+ GIC/ 45 patients		Cumulative rates at 12 months-88.7% for the occlusal surfaces and 58.8% for the palatal surfaces	
Abdellatif HM et al., 2021 Saudi Arabia [51]	3–8 years	Primary teeth	12 months	38% SDF(2×/year) 27 patients 82 carious lesions	ART—cavity restored with GIC + Protective varnish 26 patients/85 carious lesions	At 6 months: 100% At 12 months: 99%	At 6 months: 96% At 12 months: 94%
Turton B. et al., 2021 Cambodia [11]	3–11 years	At least one active decay in deciduous teeth without pulp exposure	12 months	Group 1: 38% SDF/(2× per year)/66 patients Group 2: 38% SDF + KI/(2× per year)/ 110 patients	Group 3: AgF/85 children Group 4: AgF + KI/ 57 children	Group 1: 77.3% Group 2: 65.4%	Group 3: 75.3% Group 4: 51.2%
Gao Sherry Shiqian et al., 2020 Hong Kong [43]	3–4 years	Primary teeth with ECC	30 months	38% SDF/500 patients	25% AgNO_3_ +5% NaF/495 patients	At 12 months: 60% At 30 months: 68.9%	At 12 months: 62.4% At 30 months: 70.6%
Jiang M. et al., 2020 Hong Kong [8]	3–4 years	Primary teeth	24 months	38% SDF (10 s) +GIC restorations (10 weeks after)/ 88 patients	Tonic water as Placebo +GIC restorations (10 weeks after)/84 patients	57.8% at 6 months 46.3% at 12 months 37.2% at 18 months 28.8% at 24 months	60.3% at 6 months 48.4% at 12 months 33.8% at 18 months 26.9% at 24 months
Mabangkhru S. et al., 2020 Thailand [47]	1–3 years	Primary teeth	12 months	38% SDF (10 s)/ 130 patients	5% NaF Varnish/133 patients	35.7%	20.9%
Pisarnturakit P. et Detsomboonrat P., 2020 Thailand [48]	3–5 years	Primary teeth	24 months	HRI Group: 38% SDF +Fluoride Varnish every 6 months	HRB Group LRB Group/ No intervention	Percentage of new caries in HRI group increased from 40% (at 6 months) to 65.7% (at 24 months).	Percentage of new caries in HRB group increased from 45.9% (at 6 months) to 75% (at 24 months).
Vollú AL et al., 2019 Brazil [55]	2–5 years	Occlusal decays on the primary molars	12 months	30% SDF (3 min./2×/year)/34 patients	ART with GIC /33 patients	84.6%	82.7%
Tirupathi S. et al., 2019 India [52]	6–10 years	Primary molars	12 months	38% SDF (1/year)/24 patients	5% NSSF (1×/year)/ 23 patients	71.05%	77%
Duangthip D. et al., 2018 Hong Kong [44]	3–4 years	Primary teeth	30 months	Group 1: 30% SDF (1×/year)/101 patients Group 2:30% SDF (3 applications at weekly intervals/year)/102 patients	Group 3: 5% NaF Varnish (3 applications at weekly intervals/year)	Group 1: 45% Group 2: 44%	Group 3: 51% ICDAS: 3–4
Fung MHT et al., 2018 Hong Kong [45]	3–4 years	Primary teeth	30 months	Group 1: 12% SDF (1×/year)/198 patients Group 2: 12% SDF (2×/year)/203 patients Group 3: 38% SDF (1×/year)/202 patients Group 4:38% SDF (2×/year)/196 patients		Group 1: 55.2% Group 2: 58.6% Group 3: 66.9% Group 4: 75.7%	
Monse B. et al., 2012 Filipine [54]	6–8 years	First permanentmolars: without dentinal carious lesions visible at the occlusal surface	18 months	38% SDF with tannic acid to precipitate silver (1×/year)/91 patients 38% SDF (1×/year)/ 139 patients	ART sealant: 90 patients No Treatment: 45 patients	91% 87.5%	99% 93.8%
Zhi QH et al., 2012 China [50]	3–4 years	Primary teeth	24 months	Group 1: 38% SDF (1×/year)/91 patients Group 2: 38% SDF (2×/year)/ 59 patients	Group 3: GIC	Group 1: 79.2% Group 2: 90.7%	Group 3: 81.8%
Liu BY et al., 2012 Hong Kong [46]	9 years	First permanent molar	24 months	38% SDF (1×/year)/ 121 patients	Resin Sealant/ 121 patients 5% NaF Varnish (2×/year)/ 116 patients Placebo-Water (1×/year)/ 124 patients	87.8%	Sealant Group: 92.6% NaF Group: 87.2% Placebo: 83.1%
Dos Santos VE Jr et al., 2012 Brazil [56]	5–6 years	Primary molars	12 months	30% SDF/48 patients	IRT with GIC/43 patients	66.9%	38.6%
Joshi et al., 2023 India [53]	2–6 years	Primary teeth	1, 3, 6 months	24 patients	SDF	96%	
Duangthip et al., 2016 Hong Kong [64]	3–4 years	Primary teeth	18 months	304 patients: Group 1 receives 30% silver diamine fluoride (SDF) annually, Group 2 undergoes three weekly applications of 30% SDF	Group 3 three weekly applications of 5% sodium fluoride (NaF) varnish.	Both SDF protocols are more effective than intensive NaF varnish applications in stopping caries	
Antonioni et al., 2019 United States [61]	2–17 years	Primary and permanent teeth		582	SDF		

## Abbreviations

AAPD: American Academy of Pediatric Dentistry; ART: atraumatic restorative treatment; DMFT: decayed, missed, filled teeth; ECC: early childhood caries; GIC: glass ionomer cement; HRB: high risk basic; HRI: high risk intensive; ICDAS: International Caries Detection and Assessment System; IRT: Interim Therapeutic Restoration; LRB: low risk basic; NSSF: Nano-silver-incorporated Sodium fluoride; SDF: silver diamine fluoride; SMART: silver-modified atraumatic restorative treatment.
